# Using Catalysis to Drive Chemistry Away from Equilibrium: Relating Kinetic Asymmetry, Power Strokes, and the Curtin–Hammett Principle in Brownian Ratchets

**DOI:** 10.1021/jacs.2c08723

**Published:** 2022-10-26

**Authors:** Shuntaro Amano, Massimiliano Esposito, Elisabeth Kreidt, David A. Leigh, Emanuele Penocchio, Benjamin M. W. Roberts

**Affiliations:** † Department of Chemistry, University of Manchester, Oxford Road, ManchesterM13 9PL, United Kingdom; ‡ Institute of Supramolecular Science and Engineering (ISIS), University of Strasbourg, 67000Strasbourg, France; § Department of Physics and Materials Science, University of Luxembourg, avenue de la Faïencerie, 1511Luxembourg City, G.D. Luxembourg; ∥ Department of Chemistry and Chemical Biology, University of Dortmund, Otto-Hahn-Str. 6, 44227Dortmund, Germany; ⊥ Department of Chemistry, Northwestern University, Evanston, Illinois60208, United States

## Abstract

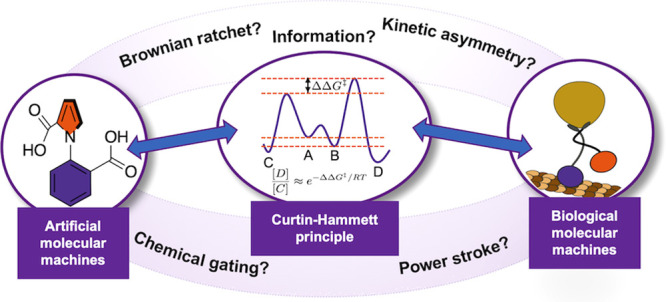

Chemically fueled autonomous molecular machines are catalysis-driven systems governed by Brownian information ratchet mechanisms. One fundamental principle behind their operation is kinetic asymmetry, which quantifies the directionality of molecular motors. However, it is difficult for synthetic chemists to apply this concept to molecular design because kinetic asymmetry is usually introduced in abstract mathematical terms involving experimentally inaccessible parameters. Furthermore, two seemingly contradictory mechanisms have been proposed for chemically driven autonomous molecular machines: Brownian ratchet and power stroke mechanisms. This Perspective addresses both these issues, providing accessible and experimentally useful design principles for catalysis-driven molecular machinery. We relate kinetic asymmetry to the Curtin–Hammett principle using a synthetic rotary motor and a kinesin walker as illustrative examples. Our approach describes these molecular motors in terms of the Brownian ratchet mechanism but pinpoints both chemical gating and power strokes as tunable design elements that can affect kinetic asymmetry. We explain why this approach to kinetic asymmetry is consistent with previous ones and outline conditions where power strokes can be useful design elements. Finally, we discuss the role of information, a concept used with different meanings in the literature. We hope that this Perspective will be accessible to a broad range of chemists, clarifying the parameters that can be usefully controlled in the design and synthesis of molecular machines and related systems. It may also aid a more comprehensive and interdisciplinary understanding of biomolecular machinery.

## Introduction

**Figure 1:**
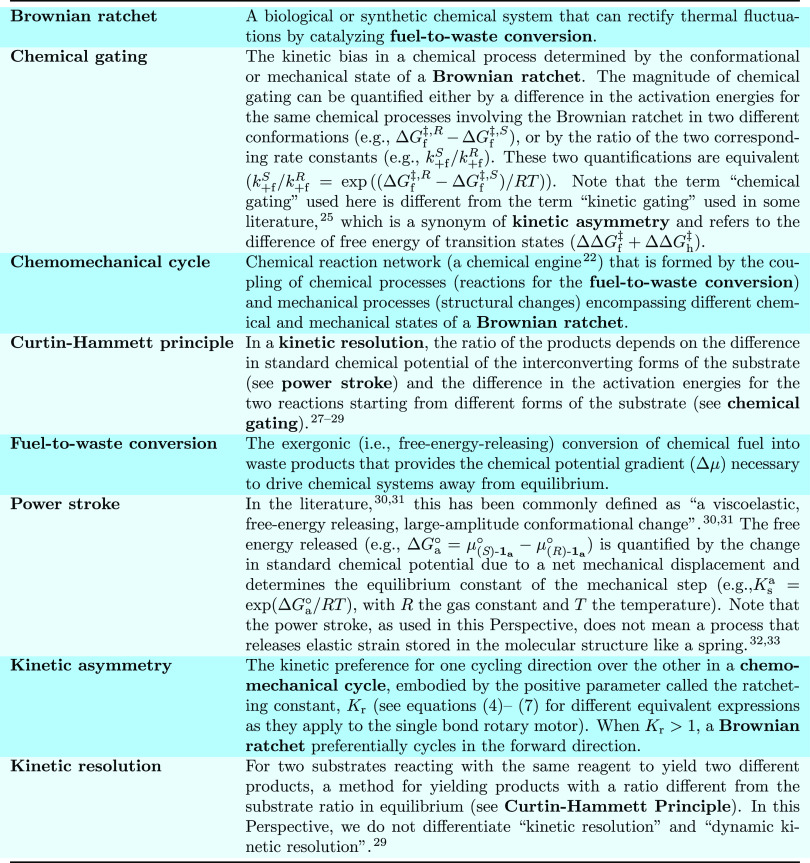
Definitions of key concepts as used in this Perspective. Terms in bold are explicitly defined. Specific examples, when provided, always refer to the anhydride formation reaction in the single-bond rotary motor fueled by carbodiimide hydration (Figure 2).

In order to solve problems as fundamental as directional movement and energy management on the molecular scale, nature has developed a myriad of molecular machinery,^1^ including much-studied examples such as kinesin^2^ and ATP synthase.^3^ Biologists,^4,5^ chemists,^6,7^ and physicists^8−11^ have all striven to understand biomolecular machines, and the first synthetic chemically driven autonomous molecular motors have also been prepared.^12−16^ A significant challenge in the understanding and design of molecular machines is that they cannot work through miniaturized mechanisms of their macroscopic counterparts, as physics is governed by different principles at the nanoscale.^17−20^ In particular, at small length scales Brownian motion and electromagnetic interactions take over from inertia and gravity in determining the dynamics of objects. As a result, motion at the molecular scale cannot be initiated in a directional and precisely controlled way using Newtonian mechanics. Instead, strategies to statistically bias the unavoidable Brownian motion must be used to achieve motion in a particular direction.^18,20^ Molecular systems implementing such strategies are often called Brownian ratchets, a terminology we will adopt throughout this Perspective (see definitions in Figure 1).^19,20^ Compared to biological Brownian ratchets, the synthetic examples of autonomous chemically fueled molecular motors^12,13,15^ and pumps^21^ reported to date are relatively simple systems. Nevertheless, they demonstrate the fundamental roles of catalysis^22^ and kinetic asymmetry^7,22−25^ (Figure 1) in obtaining directed motion. Autonomous Brownian ratchets harvest the free energy necessary for their out-of-equilibrium functioning by catalyzing fuel-to-waste conversion (Figure 1).^22,26^ To turn this into directional motion, kinetic asymmetry is required to provide a kinetic preference to move directionally while progressing around the chemomechanical cycle (Figure 1).^22,25^

Since the same fundamental principles of physics and chemistry apply to both biological and synthetic molecular machines, an understanding of biomolecular machinery can aid the design of artificial examples, while synthetic molecular motors can be seen as simplified model systems that help the understanding of their much more complex natural counterparts. Similarly, aspects of molecular machinery revealed from different viewpoints can greatly benefit each other. Unfortunately, the exchange of information between different disciplines has been hindered by the use of frameworks for molecular machinery that, superficially at least, appear to be incompatible. This is exacerbated by some classical problems of interdisciplinary research, such as the use of different scientific languages and vocabularies. For instance, the concept of kinetic asymmetry is either taken for granted in theoretical analyses or introduced in mathematical terms that are often inaccessible to synthetic chemists and biologists. Moreover, to directly apply kinetic asymmetry based on its common mathematical formulation, one would need to be able to experimentally determine the rate constants of extremely rare events such as machine-catalyzed fuel regeneration. Recently, we have made efforts to connect concepts and relationships between different disciplines in this field,^34^ but still there are seemingly contradicting analyses in the literature. For example, in biophysics, power strokes (Figure 1) are often described as important contributors to directionality.^30,33,35^ The “transient generation of a large free energy gradient so that forward motion occurs in a nearly irreversible manner”, as described by Hwang and Karplus,^33^ is sometimes considered to be the driving impetus for a motor, with the chemical transitions only used to supply energy for the process. The converse view, put forward by Astumian,^31^ considers power strokes to be irrelevant for the fundamental working principles of catalysis-driven molecular machines.

The core of this Perspective is dedicated to a resolution of this dichotomy. To make the concept of kinetic asymmetry more accessible, we first explain the intimate connection between kinetic asymmetry and the Curtin–Hammett principle,^27,28^ a well-established concept in organic chemistry that the outcome of a kinetic resolution is determined by the relative chemical potentials of interconverting starting materials as well as the relative activation energy of a reaction under kinetic control (Figure 1).^29^ Indeed, while autonomous chemically fueled artificial molecular motors and pumps are relatively new synthetic achievements, it has been recognized for some time that the key step to realizing these devices is effectively a kinetic resolution.^22,36,37^ Here we illustrate this connection using a synthetic single-bond rotary motor^13^ and a model of a kinesin walker^38^ as examples. In doing so, we show how directed motion can be induced in a molecular machine by turning a Curtin–Hammett-type resolution into a complete reaction cycle, thus de facto implementing kinetic asymmetry. By recognizing that the relevant factors for the Curtin–Hammett principle are analogous to the power stroke and chemical gating (Figure 1) in molecular machines, we derive a mathematical expression probing kinetic asymmetry in which only controllable and measurable parameters appear. The expression can be shown to be equivalent to previous ones using simple algebra and thermodynamic constraints. We detail such a proof in the Supporting Information (SI) but illustrate its main outcomes in the main body of the Perspective. Crucially, the magnitude of the power stroke appears as an experimentally accessible parameter in the new expression, replacing experimentally inaccessible rate constants associated with rare events. We then propose a solution to the debate on the role of power strokes in Brownian ratchets by showing the circumstances under which the variation of power strokes can (like other conformational changes) affect kinetic asymmetry. We conclude by discussing the role of another theoretical concept, namely, information, which is often used with different meanings in the literature of Brownian ratchets.^20,25,34,39,40^

## Brownian Ratchets and Kinetic Resolutions

**Figure 2:**
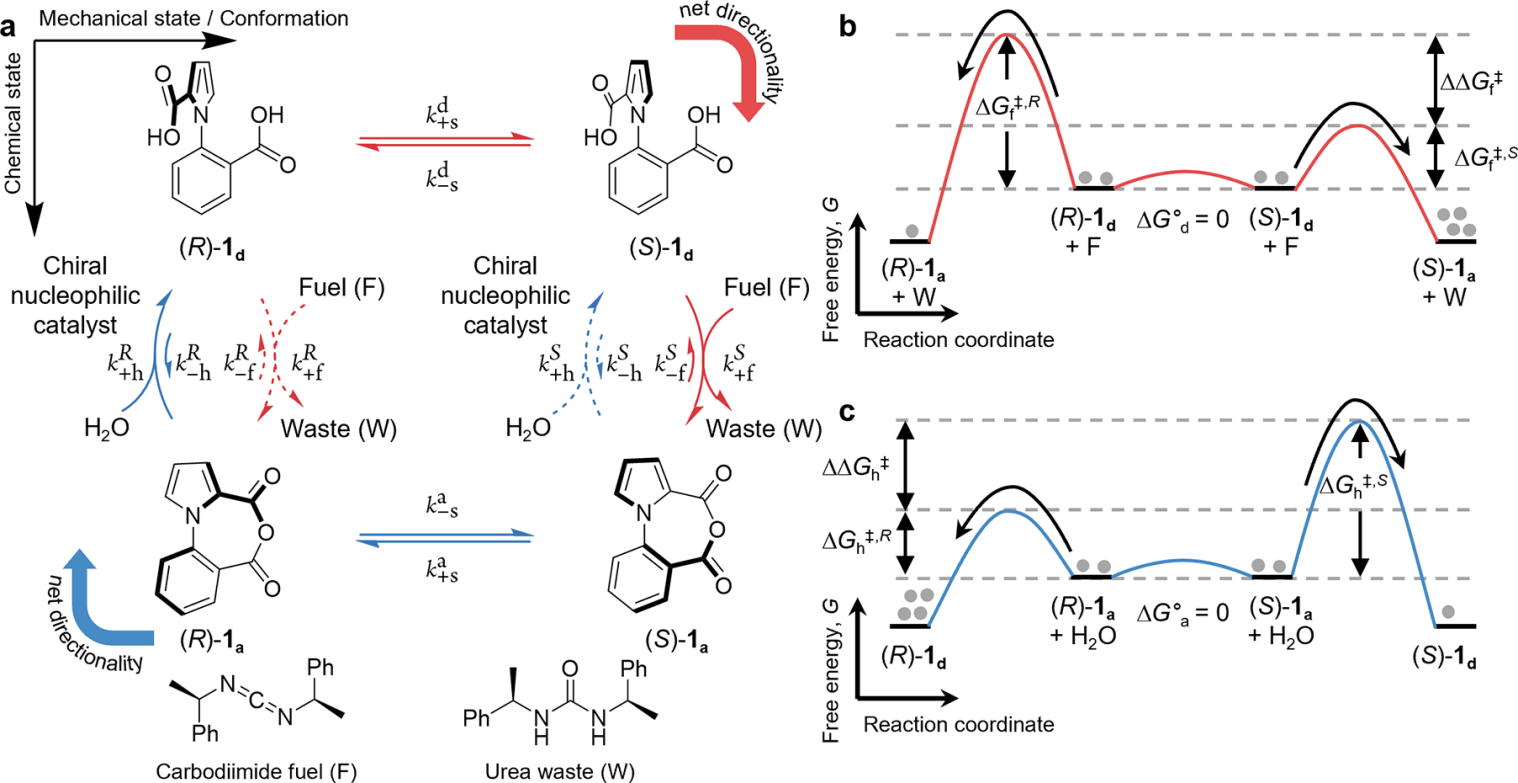
Carbodiimide-fueled single-bond rotary motor. (a) Chemomechanical cycle for motor molecule **1**. The horizontal and vertical directions show mechanical (conformational) and chemical transitions, respectively. (*S*) and (*R*) indicate the axial stereochemistry of the conformations of the motor around the C–N bond. Subscripts “d” and “a” in the species labels show the chemical states, which are the diacid (d) and the anhydride (a) forms of the motor. In the rate constants of the chemical processes (vertical transitions), superscripts “*S*” and “*R*” indicate whether a reaction involves the motor in the *S* or *R* conformation, respectively. Subscripts “f” and “h” indicate the anhydride formation and hydrolysis reactions, respectively. In the rate constants of the mechanical processes (horizontal transitions), superscripts “d” and “a” indicate whether a reaction involves the motor in the diacid or in the anhydride form, respectively. The subscript “s” stands for “stepping”. In all of the rate constants, + and – show the forward and backward reactions, respectively. Processes shown in red and blue indicate the kinetic resolution in the anhydride formation and hydrolysis reactions, respectively. Dashed arrows indicate the kinetically unfavored processes of the two competing chemical reactions. The anhydride formation reaction occurs faster from (*S*)-**1**_**d**_ than from (*R*)-**1**_**d**_ because the chiral carbodiimide fuel forms a more stable transition state (i.e., with lower activation energy) with rotamer (*S*)-**1**_**d**_. The hydrolysis occurs faster from (*R*)-**1**_**a**_ than from (*S*)-**1**_**a**_ because the chiral nucleophilic catalyst forms a more stable transition state with conformer (*R*)-**1**_**a**_. Consequently, under continuous fuel-to-waste turnover, the motor rotates in the clockwise direction, as indicated by the curved red and blue arrows at the corners, with *F*_C–H_ = 2.4 (see the text). (b) Kinetic resolution in the anhydride formation reaction. The anhydride formation reaction from (*S*)-**1**_**d**_ occurs faster than that from (*R*)-**1**_**d**_ because the activation energy for the former (Δ*G*_f_^⧧,*S*^) is smaller than the latter (Δ*G*_f_^⧧,*R*^). Δ*G*_d_^°^ = μ_(*R*)-**1**_**d**__^°^ – μ_(*S*)-**1**_**d**__^°^ is the difference of the standard chemical potentials of (*R*)-**1**_**d**_ and (*S*)-**1**_**d**_, which is zero in this motor. ΔΔ*G*_f_^⧧^ = Δ*G*_f_^⧧,*R*^ – Δ*G*_f_^⧧,*S*^ + Δ*G*_d_^°^ is the free energy difference of the two transition states for competing reaction paths. (c) Kinetic resolution in the hydrolysis step. The hydrolysis reaction from (*R*)-**1**_**a**_ occurs faster than that from (*S*)-**1**_**a**_ because the activation energy for the former (Δ*G*_h_^⧧,*R*^) is smaller than the latter (Δ*G*_h_^⧧,*S*^). Δ*G*_a_^°^ = μ_(*S*)-**1**_**a**__^°^ – μ_(*R*)-**1**_**a**__^°^ is the difference of the standard chemical potentials of (*S*)-**1**_**a**_ and (*R*)-**1**_**a**_, which is zero in this motor. ΔΔ*G*_h_^⧧^ = Δ*G*_h_^⧧,*S*^ – Δ*G*_h_^⧧,*R*^ + Δ*G*_a_^°^ is the free energy difference of the two transition states for competing reaction paths.

The role of kinetic resolutions in Brownian ratchets is well-illustrated by consideration of a recently reported autonomous single-bond rotary motor fueled by carbodiimide hydration, the simplest chemically fueled Brownian ratchet described to date (Figure 2).^13^ The motor comprises a biaryl dicarboxylic acid, which is cyclized to form an intramolecular anhydride through reaction with a carbodiimide fuel. Water present in the reaction mixture enables the subsequent hydrolysis of the anhydride to complete a chemical cycle, thereby acting as a second fuel component. The carbonyl groups can rotate past each other only in the anhydride form, yielding enantiomeric conformers (*S*)-**1**_**a**_ and (*R*)-**1**_**a**_, while rotation of the carbonyls past the *ortho*-hydrogen substituents is possible only for the diacid, yielding rotamers (*S*)-**1**_**d**_ and (*R*)-**1**_**d**_. Overall, the motor’s operation results in the chemomechanical cycle (Figure 1) depicted in Figure 2a. From a thermodynamic viewpoint, the motor is powered by catalyzing the fuel-to-waste conversion of carbodiimide (F) and water (H_2_O) to urea (waste, W), which we will consider to be chemostatted (i.e., the concentrations are kept fixed, as if by continuous addition of reagents and removal of waste products) in order to provide a constant chemical potential gradient Δμ = μ_F_ + μ_H_2_O_ – μ_W_. From a kinetic viewpoint, both chemical steps can act as kinetic resolutions, making the motor “doubly kinetically gated”.^14^ Indeed, anhydride formation can be biased using a chiral carbodiimide fuel that reacts more quickly (i.e., with a lower activation energy) with one rotamer than the other, biasing the direction of rotation in the diacid (red path in Figure 2b). Anhydride hydrolysis can be controlled with a chiral nucleophilic catalyst selecting the direction of rotation (actually a ring flip) that reacts faster with one enantiomeric conformer of the anhydride than the other (blue path in Figure 2c). The kinetic preference for undergoing a certain reaction more quickly in one conformation than another is termed chemical gating (Figure 1).

The distribution of products arising from a kinetic resolution reaction can be rationalized through the Curtin–Hammett principle,^27−29^ a well-established concept in organic chemistry (Figure 1). It states that the ratio of products of a kinetic resolution is determined by the equilibrium ratio of the rapidly exchanging starting materials multiplied by the ratio of the rate constants for each of the starting materials reacting to form the corresponding product. While the original principle^27^ was developed to deal with reactions that progress to completion under kinetic control, the idea can be readily adapted to analyze Brownian ratchets. The key difference between a traditional kinetic resolution and the operation of a Brownian ratchet is that the latter involves a cyclic process in which the initial motor state is repeatedly dynamically regenerated.

The chemomechanical cycle of the single-bond rotary motor can be conceptually split into two Curtin–Hammett-like steps, as both carbodiimide-driven anhydride formation (red path in Figure 2) and anhydride hydrolysis (blue path in Figure 2) are kinetic resolutions, with the product of one reaction being the starting material for the other. In the context of Brownian ratchets, one can apply the Curtin–Hammett principle to each of the two paths to determine the direction toward which it biases the rotation. Consider the motor in its diacid form interconverting rapidly between the two rotamers (*R*)-**1**_**d**_ and (*S*)-**1**_**d**_ according to the equilibrium constant *K*_s_^d^ = exp(Δ*G*_d_^°^/*RT*), in which *R* and *T* are the gas constant and the absolute temperature, respectively. The free energy difference Δ*G*_d_^°^ between rotamers (*R*)-**1**_**d**_ and (*S*)-**1**_**d**_ quantifies the magnitude of the so-called power stroke in the clockwise direction, namely, a free-energy-releasing mechanical step (Figure 1). Upon addition of chiral carbodiimide fuel, according to the Curtin–Hammett principle there would be a preference for transient reaction clockwise with respect to counterclockwise in the square scheme (Figure 2a), i.e., preferentially yielding anhydride (*S*)-**1**_**a**_ instead of (*R*)-**1**_**a**_, if the following condition is met:
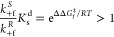
1where the Eyring equation (e.g., *k*_+f_^*S*^ = exp(−Δ*G*_f_^⧧,*S*^/*RT*) × *k*_B_*T*/*h*, where *k*_B_ and *h* are Boltzmann’s constant and Planck’s constant, respectively) connects rate constants to the corresponding Gibbs energies of activation and ΔΔ*G*_f_^⧧^ = Δ*G*_f_^⧧,*R*^ – Δ*G*_f_^⧧,*S*^ + Δ*G*_d_^°^ according to the energy profile in Figure 2b. The ratio of the two forward rate constants for the anhydride formation reactions in eq 1, *k*_+f_^*S*^/*k*_+f_^*R*^, quantifies the chemical gating afforded by this process and controls the preferred direction together with the equilibrium constant. In the original experimental conditions, the equilibrium constant *K*_s_^d^ is equal to 1 (corresponding to a null power stroke, Δ*G*_d_^°^ = 0) and therefore, the directional bias is solely dictated by the chemical gating (*k*_+f_^*S*^ > *k*_+f_^*R*^) afforded by the chiral carbodiimide fuel lowering the activation energy for anhydride formation when the substrate is (*S*)-**1**_**d**_ (Δ*G*_f_^⧧,*S*^ < Δ*G*_f_^⧧,*R*^). Similarly, when the motor is in rapid equilibrium between its anhydride conformers (*S*)-**1**_**a**_ and (*R*)-**1**_**a**_ according to the equilibrium constant *K*_s_^a^ = exp(Δ*G*_a_^°^/*RT*), “clockwise” hydrolysis will occur more rapidly, i.e., preferentially yielding diacid (*R*)-**1**_**d**_ over (*S*)-**1**_**d**_, if the following condition is met:
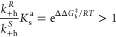
2where ΔΔ*G*_h_^⧧^ = Δ*G*_h_^⧧,*S*^ – Δ*G*_h_^⧧,*R*^ + Δ*G*_a_^°^ according to the energy profile in Figure 2c. Again, under the original experimental conditions the equilibrium constant *K*_s_^a^ is equal to 1 (Δ*G*_a_^°^ = 0), and the directional bias is dictated by the chemical gating (*k*_+h_^*R*^ > *k*_+h_^*S*^) afforded by the chiral nucleophilic catalyst lowering the activation energy for hydrolysis when the substrate is (*R*)-**1**_**a**_ (Δ*G*_h_^⧧,*R*^ < Δ*G*_h_^⧧,*S*^).

The rationale behind eqs 1 and 2, which corresponds to the Curtin–Hammett principle, is explained in SI section 1.1. We can multiply the terms in eqs 1 and 2 to define a quantity that we can call the “Curtin–Hammett asymmetry factor” (*F*_C–H_), which determines the overall directional bias in the motor’s rotary motion:
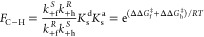
3Consistent with the Curtin–Hammett principle as it applies to the anhydride formation and hydrolysis paths considered separately, one might expect that the motor will preferentially cycle clockwise if *F*_C–H_ > 1, as is indeed the case for the original experiment (where *F*_C–H_ = 2.4),^13^ anticlockwise if *F*_C–H_ < 1, and without a preferred direction when *F*_C–H_ = 1. Such a heuristic scenario is appealing, as it implies that the directionality of a molecular motor can be determined from experimentally accessible parameters, namely, the equilibrium constants of the conformational interconversions (related to power strokes) and the chemical gating in the forward chemical reactions. It also demonstrates the role of kinetic resolution as a necessary design element, pinpointing the interplay of both chemical gating and equilibrium constants in determining the direction of rotation. However, caution is needed to ascertain whether *F*_C–H_ is a valid determinator of directionality for Brownian ratchets under all circumstances. The Curtin–Hammett principle is valid only in regimes where the backward anhydride formation (with rate constants *k*_–f_^*R*^ and *k*_–f_^*S*^) and the backward hydrolysis (with rate constants *k*_–h_^*R*^ and *k*_–h_^*S*^) can be neglected, thereby considering both steps as irreversible and thus disregarding microscopic reversibility.^41,42^ In practice, this approximation can reasonably be made only when the rates of these backward processes are so low compared with those of the forward processes that they are not readily measurable. It is therefore not guaranteed that conclusions about the directional bias in the two pathways, when considered separately and transiently, still hold for the chemomechanical cycle as a whole at the steady state, where the rates of the backward processes can be important, even when they are very low.^25,31^ In particular, we note that the value of *F*_C–H_ is a property of the motor that is independent of its operating regime and the fueling chemical potential gradient Δμ. Therefore, at equilibrium, where Δμ = 0 and microscopic reversibility imposes detailed balance (preventing directionally biased motion), the motor does not rotate preferentially in one direction even if *F*_C–H_ ≠ 1. All in all, the Curtin–Hammett asymmetry factor *F*_C–H_ is an intuitive and experimentally accessible quantity that predicts and determines directionality when a driving force keeps a system out of equilibrium. The *F*_C–H_ values for published information ratchets include 4 for a catenane-based motor,^12^ 42 for a rotaxane-based pump,^21^ 31 for a rotaxane ratchet,^16^ and 2.4 for the biaryl motor shown in Figure 2.^13^ The basis of the utility of *F*_C–H_ is mathematically justified in the next section.

## Kinetic Asymmetry and the Curtin–Hammett Principle

**Figure 3:**
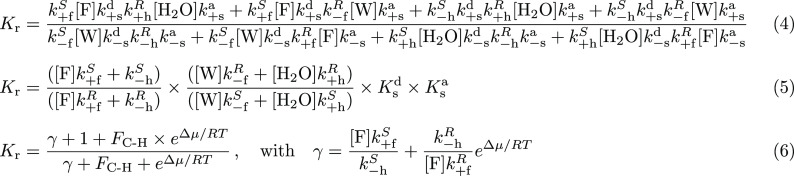
Three equivalent expressions for the ratcheting constant (*K*_r_). The equations show how *K*_r_ can be related to the Curtin–Hammett asymmetry factor (*F*_C–H_), an experimentally accessible quantity for determining the directionality of Brownian ratchets. See SI section 2 for the derivation.

As has been shown in several earlier contributions,^23−25,43−47^ a rigorous way to assess directionality in a chemomechanical cycle akin to the motor-molecule scheme in Figure 2a is to evaluate its kinetic asymmetry,^23,24^ embodied by a single parameter that is often called the ratcheting constant, *K*_r_.^48,49^ Analogous to an equilibrium constant quantifying the bias in the concentrations of reactants and products at equilibrium, *K*_r_ functions as a *nonequilibrium* constant that quantifies the bias toward traveling clockwise around the chemomechanical cycle with respect to counterclockwise in the steady state. It is the ratio of the average clockwise rotation frequency of the motor to the counterclockwise one. Following Hill’s approach,^50−52^ such a ratio can be evaluated by dividing the sum of the products of the forward rates along each possible clockwise cycle by the sum of the products of the corresponding backward rates,^46,47^ yielding eq 4 in Figure 3 (where the factor [(*S*)-**1**_**d**_][(*S*)-**1**_**a**_][(*R*)-**1**_**a**_][(*R*)-**1**_**d**_] involving the concentrations of the motor’s species has been canceled out, as it is common to all of the terms). By definition, whenever *K*_r_ > 1 the motor preferentially rotates clockwise, while counterclockwise rotation dominates when *K*_r_ < 1 and no net directionality arises when *K*_r_ = 1 (e.g., at equilibrium). By collecting common factors in eq 4, we can deduce eq 5 in Figure 3. The main difference between eqs 3 and eq 5 is that the latter incorporates the effects of all of the rate constants in the network and of the concentrations of chemostatted species. It can also be considered an extension of eq 3 when accounting for microscopic reversibility, as shown in SI section 1.2. At first sight, only under those conditions where the rate constants for backward anhydride formation (*k*_–f_^*S*^ and *k*_–f_^*R*^ in eq 5) and backward hydrolysis (*k*_–h_^*S*^ and *k*_–h_^*R*^ in eq 5) can be neglected do we obtain *K*_r_ ≈ *F*_C–H_. However, due to thermodynamic constraints, the connection between the two quantities is much stronger than that. Indeed, as formally shown in SI section 2, by imposing thermodynamic consistency on the rate constants of the network, eq 5 can be rewritten in a rather more insightful way for our purpose here, namely, as eq 6 in Figure 3, where the symbol γ is introduced to simplify the appearance of the expression and provide intuition as to how *K*_r_ varies. Equation 6 explicitly shows that when there is no driving force (Δμ = 0), kinetic asymmetry always disappears (*K*_r_ = 1), as the only possible stationary state is an equilibrium one. This reiterates that directionality can only emerge if the system is brought out-of-equilibrium by an energy source, in this case the chemical potential gradient, Δμ, between the chemostatted species. Equation 6 also proves that if the driving force is not null (Δμ > 0), the cycling direction of the motor is controlled by the Curtin–Hammett asymmetry factor *F*_C–H_, as anticipated in the previous section (also see SI eq 15). It should be noted that this property holds regardless of whether the backward reactions are negligibly slow, making the prediction of directionality by *F*_C–H_ applicable to the design of any chemically driven autonomous molecular motor. This result substantiates the intimate connection between kinetic asymmetry and the Curtin–Hammett principle: directed motion can be induced in a molecular machine by turning two Curtin–Hammett-type kinetic resolutions into a complete reaction cycle, thus de facto implementing kinetic asymmetry. We note that expressions for *K*_r_ similar to eq 6 have been previously obtained by Astumian^31,46^ and that *F*_C–H_ is equivalent to the “*q*” value in that analysis (also see SI eq 14). However, the approach presented here establishes the connection to the Curtin–Hammett principle, and the equation is derived in the form of experimentally measurable parameters.

## Power Strokes in Brownian Ratchets

**Figure 4:**
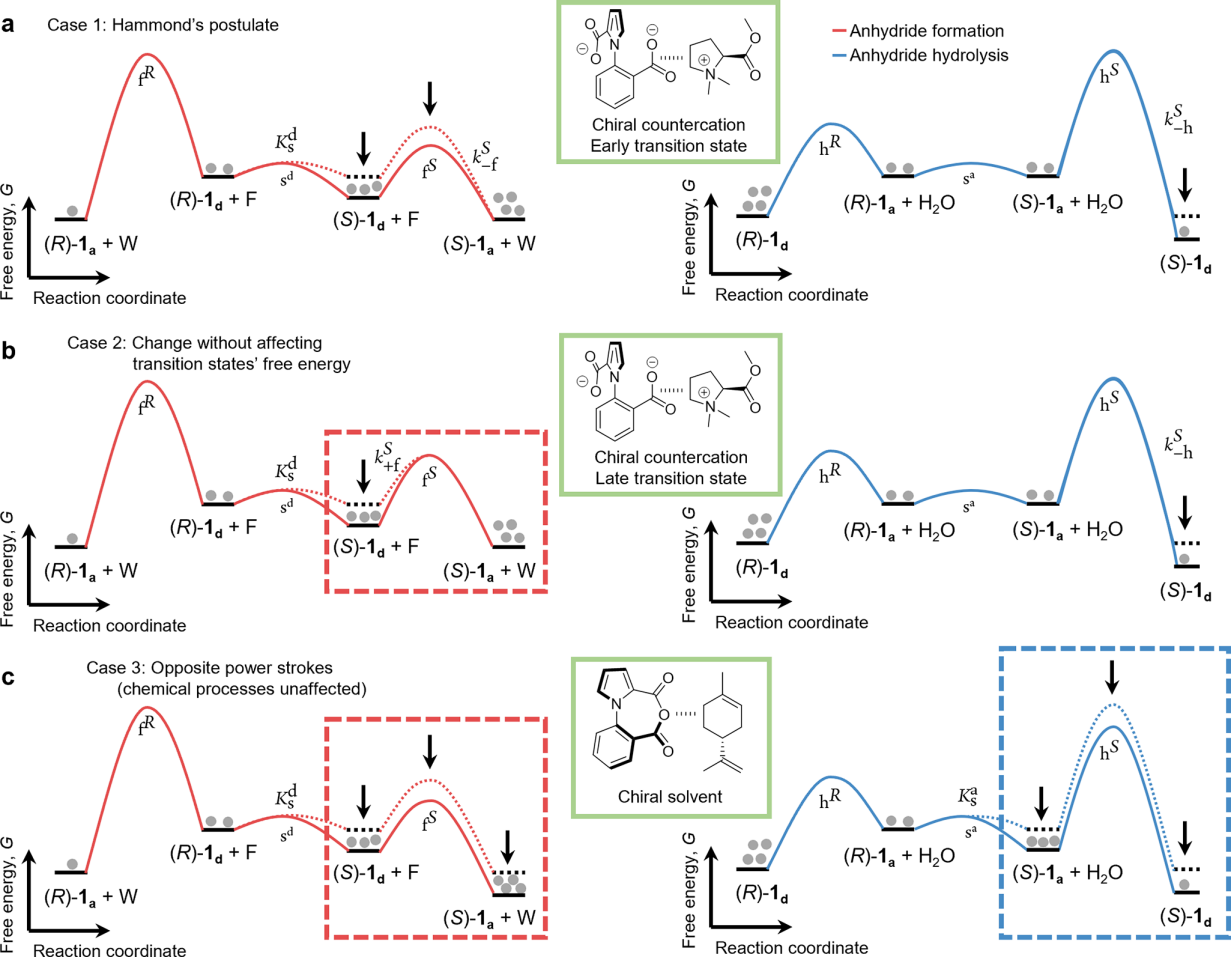
Changes in the free energy profiles for three different Brownian ratchet scenarios. Dotted and solid lines indicate the free energy profiles before and after the modification, respectively. Dashed boxes in (b) and (c) highlight the differences with respect to (a). Downward arrows indicate where free energies are varied. As a consequence of free energy variations, the values of the rate constants and equilibrium constants explicitly shown in the figure are affected. The red and blue energy profiles refer to the anhydride formation and hydrolysis reactions, respectively. (a) Case 1: Hammond’s postulate. The free energy of (*S*)-**1**_**d**_ and the transition state for the corresponding anhydride formation reaction vary. Consequently, the rate constants *k*_–f_^*S*^ and *k*_–h_^*S*^ and the equilibrium constant *K*_s_^d^ are affected. Such a change could be implemented by, for example, the use of a chiral countercation. To realize the change shown here, the transition state of the rate-determining step must come early in the reaction sequence so that the transition state is similar to the reactants. (b) Case 2: change without affecting the transition states’ free energies—only the free energy of (*S*)-**1**_**d**_ varies. Consequently, the rate constants *k*_+f_^*S*^ and *k*_–h_^*S*^ and the equilibrium constant *K*_s_^d^ are affected. Such a change could be implemented by, for example, the use of a chiral countercation. To realize the change shown here, the transition state of the rate-determining step must come late in the reaction sequence so that the transition state is similar to the products. (c) Case 3: opposite power strokes (chemical processes are unaffected). The free energies of (*S*)-**1**_**d**_, (*S*)-**1**_**a**_, and the transition state between them varies. Consequently, the equilibrium constants *K*_s_^a^ and *K*_s_^d^ are affected. Such a change might be implemented by, for example, the use of a chiral solvent.

The discussion in the previous section seemingly contradicts earlier results based on kinetic models that indicate that power strokes, and therefore the values of equilibrium constants for the mechanical steps, are irrelevant for tuning the directionality of chemically driven Brownian ratchets.^31^ However, the situation is more subtle than that. Indeed, even though we have discussed circumstances where power strokes are a relevant feature for directionality (as is often considered in biophysics^30,33,35^), this does not necessarily contradict the earlier kinetic results. The viewpoint that power strokes are irrelevant in tuning the directionality of chemically driven Brownian ratchets originates from the possibility of rewriting eq 5 in forms such as the following (see SI section 2 for the derivation):
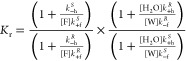
7where only the rate constants for the forward and backward anhydride formation and hydrolysis reactions appear, with no explicit role for the equilibrium constants of the mechanical reactions.^25^ Expressing *K*_r_ in this way has the effect of making the mathematics more elegant and concise, but the greater reliance on unmeasurable quantities renders it less intuitive for an experimental approach. Equations 6 and 7 distill the essence of the controversy. In the former, the magnitudes of the power strokes (Δ*G*_a_^°^ and Δ*G*_d_^°^, defining *K*_s_^a^ and *K*_s_^d^, respectively) appear in *K*_r_ through the parameter *F*_C–H_ according to eq 3, thus seemingly affecting the directionality in line with many models of biological molecular machines.^30,33,35^ In the latter, as in common expressions for the kinetic asymmetry of Brownian ratchets based on chemical kinetics,^25,31,46^ power strokes (equilibrium constants) do not appear as variables. Since eqs 6 and 7 are two mathematically equivalent expressions, one can see that determining whether varying the magnitude of power strokes in a Brownian ratchet design may affect directionality is not a clearly defined question unless particular conditions are specified (vide infra). Indeed, as made explicit in SI section 2, the reason why eqs 6 and 7 are equivalent is that the equilibrium constants of the mechanical processes and the rate constants of the chemical processes are not independent of each other due to thermodynamic constraints. Loosely speaking, this means that either can be expressed in terms of the other: how varying the magnitude of power strokes affects the kinetic asymmetry of a system depends on how the mutual dependence between the equilibrium constants and the rate constants of chemical processes manifests itself in the system. To illustrate this point, we consider three different instructive scenarios in Figure 4.

Figure 4a shows hypothetical modifications of the motor’s design and/or environment that make one rotamer more favorable than the other. In particular, it shows the effect of the addition of a chiral counterion to the carboxylate form of the diacid moiety that stabilizes rotamer (*S*)-**1**_**d**_ with respect to (*R*)-**1**_**d**_. This introduces a positive power stroke in the clockwise direction (Δ*G*_d_^°^ > 0, corresponding to *K*_s_^d^ = exp(Δ*G*_d_^°^/*RT*) > 1). However, whether this alters *K*_r_ depends on how the design modification affects the transition states of the chemical reactions and in turn their rate constants. One possibility is that the system is governed by Hammond’s postulate (or Leffler’s assumption),^53,54^ that is, the transition states of exergonic reactions (such as both anhydride formation and hydrolysis) are much more similar to the reactants than the products (i.e., they are “early” transition states). Consider the scenario depicted in Figure 4a, where the transition state of the anhydride formation reaction converting (*S*)-**1**_**d**_ into (*S*)-**1**_**a**_ gets stabilized by this design modification, while the one of the hydrolysis reaction involving (*S*)-**1**_**d**_ as a product remains unchanged. As a consequence, as shown in Figure 4a, the increase in *K*_s_^d^ is accompanied by an increase in the rate constant *k*_–f_^*S*^ for the backward anhydride formation and a decrease in the rate constant *k*_–h_^*S*^ for backward hydrolysis, while all of the other rate constants are unaffected. Crucially, in this scenario *F*_C–H_ in eq 3 increases, and both eqs 6 and 7 predict that *K*_r_ will increase as a consequence of the power-stroke-inducing design modification.

However, Hammond’s postulate does not always hold. For example, in the case of a sufficiently complex reaction mechanism there is no guarantee that the chiral counterion will interact effectively with the transition state for the rate-determining step. In this circumstance, the case opposite to the one discussed above may occur, where the transition state is not affected by the modification stabilizing the (*S*)-**1**_**d**_ rotamer with respect to (*R*)-**1**_**d**_. This second scenario is depicted in Figure 4b, which shows that if the absolute free energies of transition states remain unaffected, then introducing the power stroke leads to lowering of both the rate constant *k*_+f_^*S*^ for the forward anhydride formation and the rate constant *k*_–h_^*S*^ for the backward hydrolysis by the same amount (i.e., by a factor of exp(−Δ*G*_d_^°^/*RT*)). In such a case, *F*_C–H_ in eq 3 is unaltered, and both eqs 6 and 7 predict that *K*_r_ will not be affected.

In a third instructive scenario, we can consider a modification in the motor’s design and/or environment that stabilizes all of the motor’s structures with the same chirality by the same amount, for example, favoring the *S* state of either the diacid and anhydride over their *R* counterparts. This might be achieved using a chiral solvent that interacts differently with the different enantiomeric states but may provide a similar *S*-to-*R* bias in both the diacid and anhydride forms of the motor. As shown in Figure 4c, in such a case the transition states for both the anhydride formation and hydrolysis reactions involving *S* species are stabilized, but the corresponding activation energies (and thus the rate constants for the chemical processes) are unaltered due to the simultaneous stabilization of both the reactants and products. Only the equilibrium constants of the mechanical steps are changed. In particular, *K*_s_^d^ increases while *K*_s_^a^ decreases by the same amount. It should be noted that if the activation energies for all of the chemical steps are unchanged, the fact that the changes in the equilibrium constants compensate for each other is required for thermodynamic consistency (see SI eqs 9a and 9b). Overall, the net power stroke in this scenario is null, as the free energy gained from one mechanical transition is lost in the other. Again, *F*_C–H_ in eq 3 is unaltered, and both eqs 6 and 7 predict that *K*_r_ remains constant in this scenario.

The three scenarios discussed here are limiting (and somewhat simplified) cases of real situations, which will typically lie somewhere in between with more complex modifications of the free energy profiles. Nonetheless, their analysis in the light of eqs 6 and 7 demonstrates that the two seemingly contrasting viewpoints in the literature are actually not contradictory: on the one hand, introducing power strokes through chemical modifications into a chemomechanical cycle may enhance directionality, but on the other, that can happen only under specific kinds of functional dependences of the chemical rate constants on the mechanical equilibrium constants (e.g., when the Hammond postulate is operating), leading to a change in the Curtin–Hammett asymmetry factor *F*_C–H_. Thus, power strokes do not have a special role per se (i.e., due to the fact that they are energy-releasing steps^31^) but can have the same consequences as other processes in the chemomechanical cycle. From a theoretical perspective, what ultimately matters is how a particular design modification affects the kinetic asymmetry of the system, i.e., the differences in the absolute free energies of transition states quantified by ΔΔ*G*_f_^⧧^ + ΔΔ*G*_h_^⧧^ in eq 3. This is the reason why catalysis-driven molecular motors and related systems are governed by Brownian ratchet mechanisms. From an experimental perspective, a relevant outcome of this discussion is that it shows that the effect of certain design modifications on the directionality of a Brownian ratchet when operated out of equilibrium (i.e., whether it is enhanced, worsened, or unaltered) can be properly evaluated using a Curtin–Hammett approach by determining *F*_C–H_ via eq 3, which comprises quantities that can often be determined experimentally. We stress that, in contrast with the power stroke mechanism, determining the direction and magnitude of the power strokes is not sufficient to predict the directionality of a chemically driven motor and that care should be taken not to rely on equilibrium constants alone for motor design.

## Kinesin as a Biomolecular Motor Case Study

**Figure 5:**
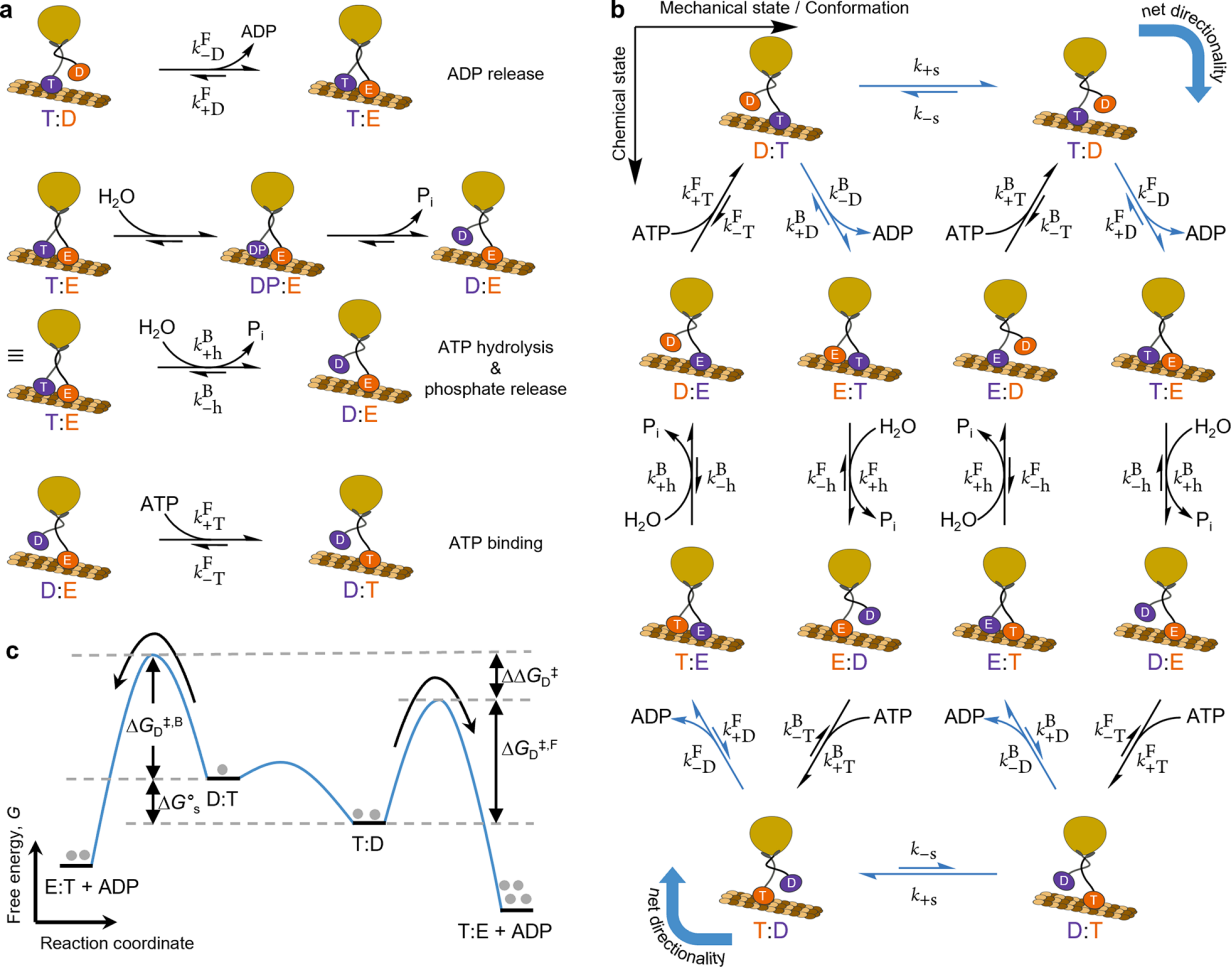
Kinesin walker. (a) Chemical reactions involving kinesin’s heads. Different states of kinesin are distinguished by the type of nucleotide attached to each head (T, ATP; D, ADP; E, empty; DP, ADP and inorganic phosphate). The heads are considered to be chemically identical but distinguishable (orange and purple in the cartoons) and are always attached to the microtuble except when they bind ADP. ADP unbinds with forward rate constant *k*_–D_^F/B^ and backward rate constant *k*_+D_^F/B^. ATP is hydrolyzed with forward rate constant *k*_+h_^F/B^ and backward rate constant *k*_–h_^F/B^, consuming water and eventually releasing inorganic phosphate P_i_. The whole process is modeled as a single coarse-grained reaction. One empty head binds ATP with forward rate constant *k*_+T_^F/B^ and backward rate constant *k*_–T_^F/B^. Superscripts F and B indicate whether the reaction happens at the front (right) or back (left) head. (b) Chemomechanical cycle for kinesin. The horizontal and vertical directions show mechanical (conformational) and chemical transitions, respectively. The rate constants *k*_+s_ and *k*_–s_ indicate forward and backward stepping, respectively. Processes shown in blue indicate the kinetic resolution in the ADP release. Under continuous fuel-to-waste turnover, kinesin moves left to right, corresponding to clockwise cycling as indicated by the curved blue arrows at the corners, with *F*_C–H_ = 1.25 × 10^6^ (see the text). (c) Kinetic resolution in the ADP release. Due to the equivalence of the two heads, the same free energy profile applies to both the top and bottom parts of the chemomechanical cycle. Δ*G*_D_^⧧,B^ and Δ*G*_D_^⧧,F^ are the activation energies for ADP release from D:T and T:D, respectively. Δ*G*_s_^°^ is the difference in the standard chemical potentials of D:T and T:D. ΔΔ*G*_D_^⧧^ = Δ*G*_D_^⧧,B^ – Δ*G*_D_^⧧,F^ + Δ*G*_s_^°^ is the free energy difference in the two transition states for competing reaction paths. Since ΔΔ*G*_D_^⧧^ is nonzero, one product (T:E) forms preferentially over the other (E:T), which leads to directional movement of kinesin.

We now turn our attention to the relevance of kinetic resolutions to biological molecular motors, as illustrated with a simple, yet paradigmatic, model of a kinesin walker.^10,31,38,40,55^ The model uses experimental evidence and considers kinesin to be composed of two “heads” attached to a cargo that walk along a microtubule through a “hand-over-hand” mechanism.^5,56−58^ The two heads are assumed to be identical but distinguishable, and only states where at least one head is attached to the microtubule are considered. A head is always attached to the microtubule, except when it binds ADP. As shown in Figure 5a, each head can undergo three different reactions that change its chemical state. When ADP is bound to a head, it can be released. Such a release is always associated with attachment of the head to the microtubule, whereas binding of ADP (the backward process) is always associated with detachment. When ATP is bound to a head, it can be hydrolyzed, consuming water and producing inorganic phosphate, P_i_, which is subsequently released. Finally, an empty head attached to the microtubule can bind one molecule of ATP. Overall, kinesin’s stochastic stride is represented by the chemomechanical cycle depicted in Figure 5b, where in addition to the chemical reactions there are two mechanical stepping reactions inverting the back and front heads (sometimes called the trailing and leading heads, respectively). The equilibrium constant for the mechanical stepping is denoted as *K*_s_ and implies a power stroke in the forward direction when *K*_s_ > 1. By traveling a full cycle in the clockwise direction, a single kinesin undergoes two steps toward the right end of the microtubule by exchanging the back and front heads twice.

As shown in SI section 3, by following the same logic used to determine the directionality of the carbodiimide-fueled single-bond rotary motor, the directional bias in the average motion of kinesin when fueled by conversion of ATP to ADP and P_i_ can be understood in terms of kinetic resolution. Indeed, the analogue of the Curtin–Hammett asymmetry factor for kinesin (see SI eq 20 in SI section 3) is
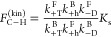
8Again, kinesin will move on average to the right when *F*_C–H_^(kin)^ > 1 and to the left when *F*_C–H_^(kin)^ < 1, while no net motion will be observed when *F*_C–H_^(kin)^ = 1. Here the chemical gatings on all three forward chemical reactions (ATP binding, ATP hydrolysis, and ADP release) contribute in principle to determine the directionality. However, it is suggested in the literature that chemical gating is not significant at the level of these reactions, implying that *k*_+T_^F^ ≈ *k*_+T_^B^, *k*_+h_^F^ ≈ *k*_+h_^B^, and *k*_–D_^F^ ≈ *k*_–D_^B^.^38,56,57^ As a consequence, *F*_C–H_ ≈ *K*_s_ ≈ 1.25 × 10^6^ according to experimental data.^38,57^ This is in line with observations made in biological motors^30,33,59^ but also implies the presence of kinetic bias in the backward chemical reactions^38^ given the equivalence between the analogs of eqs 6 and 7 (*k*_–T_^F^ ≠ *k*_–T_^B^ and/or *k*_–h_^F^ ≠ *k*_–h_^B^ and/or *k*_+D_^F^ ≠ *k*_+D_^B^; see SI eq 19). In the case of kinesin, the hydrolysis and ATP binding reactions do not involve two interconverting mechanical states as the initial states and cannot be described in terms of kinetic resolution. Still, the ADP release reaction can be represented as a kinetic resolution in which the Curtin–Hammett principle applies (Figure 5c).

## Power Stroke Mechanism versus Brownian Ratchet Mechanism?

Based on the analyses in the previous sections, we can see that the debate on the all-or-nothing role of power strokes in determining the directionality of catalysis-driven Brownian ratchets^31,33,35^ is not merited. Here we summarize how the apparent dichotomy arose in the literature and explain its resolution. The concept of the power stroke originated from the study of the mechanism of muscle contraction by myosin and was introduced to describe a large structural change in molecular motors that is responsible for the mechanical work performed by the molecular motor.^60,61^ Since its suggestion, the power stroke has been regarded as an essential element for biomolecular motors to perform work and has dominated the understanding of their mechanisms.^32,62−64^ Currently, power strokes are considered to occur by conformational changes that involve a downhill free energy gradient.^32,33^ In the 1990s, the Brownian ratchet mechanism was proposed as an alternative to the power stroke mechanism.^8,43,65−67^ The concept of the Brownian ratchet refers to a mechanism that rectifies Brownian motion to generate directional motion and perform work and can be traced back originally to Lippmann^68^ and Smoluchowski^69^ as an example of a Second Law-breaking perpetual motion machine. The concept was later more fully developed by Huxley^70^ and Feynman,^71^ who showed how such a mechanism might work if it were coupled to the dissipation of an appropriate energy source. Further investigation of the Brownian ratchet led to the development of various theoretical models, such as the flashing ratchet, the rocking ratchet, and the information ratchet (see the next section for a discussion of the role of information in Brownian ratchets).^20^ As is clear from this history, both the power stroke and Brownian ratchet mechanisms have attempted to capture the origin of directionality of molecular motors, which has led to an impression that they represent conflicting viewpoints as to the mechanism by which directional motion can be induced at the molecular level. Some attempts were made to compromise these two viewpoints by quantifying the contributions from each mechanism to the overall directionality.^4,11,30,35,72,73^ However, these still treat Brownian ratchet and power stroke mechanisms as distinct. Consequently, they were not necessarily helpful for developing a straightforward design principle for molecular motors that could be applied to the design of synthetic molecular motors.

It is more helpful and correct to consider the Brownian ratchet mechanism as the fundamental, overarching mechanism underlying catalysis-driven molecular motors. The power stroke, meanwhile, is one element that can be used to modify the directionality of Brownian ratchets, with the other element being chemical gating. We can obtain a deeper understanding about conditions under which power strokes can affect the directionality of molecular motors by considering the three different scenarios for their introduction shown in Figure 4. Furthermore, we can quantify the contributions of power strokes and chemical gating to the overall directionality using eqs 3 and 6. These insights not only settle the apparent dichotomy between the power stroke and Brownian ratchets but also can guide the design of new synthetic molecular motors. It should be noted that a power stroke is simply a type of large-amplitude conformational change and is not an essential element for directional motion as envisaged when power stroke mechanisms were originally proposed.^32,60−64^ The ability of a power stroke to affect directionality arises not from any special characteristics it has in the overall mechanism but rather from its contribution to kinetic asymmetry, the fundamental working principle of Brownian ratchets. It should also be noted that in this understanding of a Brownian ratchet, different mechanical states can have considerably different standard chemical potentials, which allows for the incorporation of power strokes. This point is worth mentioning explicitly because some references implicitly limit the concept of Brownian ratchets to systems with mechanical states having the same or very similar standard chemical potentials.^30,73,74^ Our understanding complies with the general concept of the Brownian ratchet because some variations of the Brownian ratchet, such as the flashing (pulsating) ratchet, incorporate power strokes to generate directional motion.^75,76^

## The Role of Information in Brownian Ratchets

Brownian ratchets of the kind considered in this Perspective, namely, those working autonomously in the presence of a chemical potential gradient, are often called information ratchets.^20,25,77^ The reason for this is that in order to work directionally, they must necessarily display chemical gating at the level of at least one process involved in the catalysis of the fuel-to-waste conversion, no matter whether such a gated process is an experimentally observable forward reaction or a very unlikely backward reaction (for our two examples in Figures 2 and 5, such a condition is apparent from eq 7 and SI eq 19, respectively). This implies that, at least in principle, the behavior of a Brownian ratchet is dependent on information about its current state: it will react more quickly with fuel if doing so enables forward movement or prevents backward movement. In this sense, information is encoded in the molecular structure, which is reflected in biased chemical reactivity.

Recently,^34^ we studied how the concept of an information ratchet relates to the notion of mutual information as used in information theory^78^ and information thermodynamics.^39^ In the context of Brownian ratchets, mutual information measures the statistical correlations between the chemical and mechanical states of the ratchet, answering the following question: how much information can be inferred regarding the mechanical state if only the chemical state is measured? In this sense, information is encoded in the probability of finding the ratchet in a certain chemical and mechanical state and is reflected in lower or higher entropy of the system. Furthermore, in information thermodynamics, the concept of mutual information is clearly related to that of Maxwell’s demon, a physical system in which some processes are maintained out of equilibrium by virtue of the information flow generated by other processes (a subset of processes in the system interpreted as the demon).^79^ Interestingly, it can be shown that whenever the net power stroke of its chemomechanical cycle is null, a Brownian ratchet coincides with a Maxwell’s demon in which chemical processes generate the information flow keeping the mechanical processes out of equilibrium.^34,80^ Therefore, in cases such as the carbodiimide-fueled single-bond rotary motor discussed here or the Fmoc-Cl-fueled catenane motor reported by Wilson et al.,^12^ the concept of an information ratchet is synonymous with that of a chemical Maxwell’s demon. Whenever a net power stroke is present in the chemomechanical cycle, such as in the case of kinesin, an energy flow is present in addition to the information flow, which leads to heat dissipation by the mechanical processes.^34^ In such cases, there is not always a one-to-one correspondence between information ratchets and Maxwell’s demon, and the role played by the physical notion of information may depend on the regime of operation of the ratchet.^80^

In the particular case of processive biological motors such as kinesin, a third viewpoint on the role of information that focuses on the correlation between the two heads was recently proposed.^40^

We find these kind of connections between three somewhat disparate disciplines such as synthetic chemistry, theoretical physics, and molecular biology particularly relevant and worth mentioning, not only because connecting fields of knowledge is a good thing to do per se but also because aiding the translation of concepts and relationships between different frameworks fosters a deeper and more interdisciplinary understanding of a system, which can lead to unintuitive connections and breakthroughs.

## Conclusions

In this Perspective, we have explained design principles that underpin chemically driven autonomous molecular motors by relating their mechanism to the Curtin–Hammett principle, a well-known and intuitive way of predicting the outcome of kinetic resolutions. We outline a practical determination of directionality in terms of the parameter *F*_C–H_ (the equivalent of Astumian’s “*q*” derived using trajectory thermodynamics^31,46^), which is expressed in terms of readily accessible rate constants and equilibrium constants. In doing so, we resolve some of the practical issues pertaining to kinetic asymmetry, the fundamental principle behind Brownian ratchets. These issues were (i) the difficulty of relating kinetic asymmetry analyses to readily accessible experimental data when rate constants of extremely rare events appear in the mathematical expressions and (ii) the fact that models and data suggest a role for power strokes, which were previously assumed to contradict the kinetic asymmetry principle. We have shown that directionality in Brownian ratchets can be experimentally determined just by quantifying chemical gating (i.e., activation free energy differences) and equilibrium constants (i.e., power strokes), clarifying their contributions to kinetic asymmetry. In particular, although the difference in the free energies of transitions states is the fundamental determinator of directionality in Brownian ratchets, it can also be induced through applying chemical gating and power strokes. Thus, chemically driven autonomous molecular motors can be designed by coupling a chemical reaction cycle for fuel-to-waste conversion (i.e., a catalytic cycle) with mechanical steps, with bias of the whole process achieved through the Curtin–Hammett principle to realize kinetic asymmetry.

Using the definitions of the terms in Figure 1, the concepts of power stroke and Brownian ratchet are not a dichotomy.^33^ However, it is still useful to distinguish the contributions of the different elements, such as chemical gating and power strokes, in generating directional motion through a Brownian ratchet mechanism. Indeed, information thermodynamic analysis^34^ of an autonomous catenane rotary motor^12^ revealed two contributions to free energy transduction within the motor to generate directional motion, namely, energy flow and information flow, with the former directly related to the presence of power strokes and the latter more subtly related to chemical gating.^80^

The connection between the Curtin–Hammett principle and kinetic asymmetry^22^ and the recognition that chemically driven autonomous molecular motors are catalysts for fuel decomposition^22,25,26^ should help insights from other fields (e.g., asymmetric catalysis, organocatalysis) to be used to design new autonomous molecular motors. We hope that the analysis in this Perspective will contribute to developing a unified and interdisciplinary understanding of the mechanism of synthetic and biological molecular motors as well as other phenomena for which kinetic asymmetry plays a key role, such as light-driven systems,^81−83^ driven self-assembly,^48,49^ enzyme chemotaxis,^84^ synthetic transformations,^85^ and accelerated catalysis.^86^ Such a synergy of design and understanding would be an important step forward for bioinspired molecular nanotechnology.

## Supporting Information Available

Discussion of the Curtin–Hammett principle, algebraic manipulation of the ratcheting constant *K*_r_, and *K*_r_ for kinesin (PDF)
