# Supplementary material for: Using Catalysis to Drive Chemistry Away from Equilibrium: Relating Kinetic Asymmetry, Power Strokes, and the Curtin–Hammett Principle in Brownian Ratchets
Source: J Am Chem Soc. 2022 Oct 26;144(44):20153–64. doi: 10.1021/jacs.2c08723 (PMC9650702; doi:10.1021/jacs.2c08723)
Supplement: Supplementary file 1 — ja2c08723_si_001.pdf [file ja2c08723_si_001.pdf]

Supplementary information for Using catalysis to drive chemistry away from equilibrium: Relating kinetic asymmetry, power strokes and the Curtin-Hammett principle in Brownian ratchets

Shuntaro Amano,[†,‡] Massimiliano Esposito,[¶] Elisabeth Kreidt,[†,§] David A. Leigh,[*,†] Emanuele Penocchio,[*,¶,‖] and Benjamin M. W. Roberts[†]

†*Department of Chemistry, University of Manchester, Oxford Road, Manchester, M13 9PL United Kingdom*

‡*Institute of Supramolecular Science and Engineering (ISIS), University of Strasbourg, Strasbourg, 67000 France*

¶*Department of Physics and Materials Science, University of Luxembourg, avenue de la Faïencerie, Luxembourg City, 1511 G.D. Luxembourg*

§*Department of Chemistry, Biochemistry and Inorganic Chemistry, University of Dortmund, Otto-Hahn-Str. 6, Dortmund, 44227 Germany*

‖*Department of Chemistry, Northwestern University, Evanston, 60208 Illinois (United States)*

E-mail: david.leigh@manchester.ac.uk; emanuele.penocchio@northwestern.edu

Contents

1 The Curtin-Hammett principle **2**

1.1 Standard derivation 2

1.2 From Curtin-Hammett to Kinetic Asymmetry 3

2 Algebraic manipulation of the ratcheting constant K_r **4**

3 K_r for kinesin **7**

References **9**

1 The Curtin-Hammett principle

1.1 Standard derivation

The Curtin–Hammett principle applies when two substrates in equilibrium react to form two different products.[1–3] Consider a mixture of two rotamers (S)-$\mathbf{1_d}$ and (R)-$\mathbf{1_d}$ in fast equilibrium to which an excess of the chiral carbodiimide fuel is instantaneously added, which prompts the formation of anhydrides (S)-$\mathbf{1_a}$ and (R)-$\mathbf{1_a}$ according the red path in Figure 2. As long as the anhydride formation reactions can be considered irreversible (e.g., right after the addition of fuel, when the products necessary for the backward reaction are not yet formed), the rate $J^S_{+\mathrm{f}}$ at which anhydride (S)-$\mathbf{1_a}$ will form from diacid (S)-$\mathbf{1_d}$ is given as

$$J^S_{+\mathrm{f}} = k^S_{+\mathrm{f}}[\mathrm{F}][(S)\text{-}\mathbf{1_d}]\,, \tag{1}$$

while that of (R)-$\mathbf{1_a}$ from (R)-$\mathbf{1_d}$ as

$$J^R_{+\mathrm{f}} = k^R_{+\mathrm{f}}[\mathrm{F}][(R)\text{-}\mathbf{1_d}] = k^R_{+\mathrm{f}}[\mathrm{F}] \times \frac{[(S)\text{-}\mathbf{1_d}]}{K^\mathrm{d}_\mathrm{s}}\,, \tag{2}$$

where we exploit the assumption of fast equilibration in the diacid form to express $[(R)\text{-}\mathbf{1_d}]$ as $[(S)\text{-}\mathbf{1_d}]/K_\mathrm{s}^\mathrm{d}$. From the above two equations, the bias in the tendency of the motor in its diacid form to react clockwise with respect to counterclockwise along the red path of the chemomechanical cycle in Figure 2a can be quantified by the ratio of their formation rates as

$$\frac{J_{+\mathrm{f}}^S}{J_{+\mathrm{f}}^R} = \frac{k_{+\mathrm{f}}^S}{k_{+\mathrm{f}}^R} \times K_\mathrm{s}^\mathrm{d} \,. \tag{3}$$

Similarly, the bias in the formation of rotamers $(R)\text{-}\mathbf{1_d}$ and $(S)\text{-}\mathbf{1_d}$ via the hydrolysis of a rapidly equilibrating mixture of the enantiomeric conformers $(R)\text{-}\mathbf{1_a}$ and $(S)\text{-}\mathbf{1_a}$, and therefore the tendency to react clockwise with respect to counterclockwise along the blue path in the chemomechanical cycle, can be quantified as

$$\frac{J_{+\mathrm{h}}^R}{J_{+\mathrm{h}}^S} = \frac{k_{+\mathrm{h}}^R}{k_{+\mathrm{h}}^S} \times K_\mathrm{s}^\mathrm{a} \,. \tag{4}$$

1.2 From Curtin-Hammett to Kinetic Asymmetry

In the above derivation, we isolated the red (anhydride formation) and blue (hydrolysis) paths in Figure 2a, treating them independently. However, we can repeat the same reasoning accounting for the contribution of the backward hydrolysis reaction to the kinetic resolution of rotamers $(S)\text{-}\mathbf{1_d}$ and $(R)\text{-}\mathbf{1_d}$ and of the backward anhydride formation reaction to the kinetic resolution of the enantiomeric conformers $(R)\text{-}\mathbf{1_a}$ and $(S)\text{-}\mathbf{1_a}$. By considering the former case, the rate at which anhydride $(S)\text{-}\mathbf{1_a}$ will form from diacid $(S)\text{-}\mathbf{1_d}$ will now have two contributions

$$J_{+\mathrm{f}}^S + J_{-\mathrm{h}}^S = (k_{+\mathrm{f}}^S[\mathrm{F}] + k_{-\mathrm{h}}^S)[(S)\text{-}\mathbf{1_d}] \,, \tag{5}$$

as well as that of $(R)\text{-}\mathbf{1_a}$ from $(R)\text{-}\mathbf{1_d}$

$$J_{+\mathrm{f}}^R + J_{-\mathrm{h}}^R = (k_{+\mathrm{f}}^R[\mathrm{F}] + k_{-\mathrm{h}}^R)[(R)\text{-}\mathbf{1_d}] = (k_{+\mathrm{f}}^R[\mathrm{F}] + k_{-\mathrm{h}}^R) \times \frac{[(S)\text{-}\mathbf{1_d}]}{K_\mathrm{s}^\mathrm{d}} \,, \tag{6}$$

resulting in a slightly refined expression of the kinetic bias

$$\frac{J^S_{+\mathrm{f}} + J^S_{-\mathrm{h}}}{J^R_{+\mathrm{f}} + J^R_{-\mathrm{h}}} = \frac{(k^S_{+\mathrm{f}}[\mathrm{F}] + k^S_{-\mathrm{h}})}{(k^R_{+\mathrm{f}}[\mathrm{F}] + k^R_{-\mathrm{h}})} \times K^{\mathrm{d}}_{\mathrm{s}} \,. \tag{7}$$

Analogously, the kinetic bias for the kinetic resolution of the conformers (R)-$\mathbf{1_a}$ and (S)-$\mathbf{1_a}$ becomes

$$\frac{J^R_{+\mathrm{h}} + J^R_{-\mathrm{f}}}{J^S_{+\mathrm{h}} + J^S_{-\mathrm{f}}} = \frac{(k^R_{+\mathrm{h}}[\mathrm{H_2O}] + k^R_{-\mathrm{f}}[\mathrm{W}])}{(k^S_{+\mathrm{h}}[\mathrm{H_2O}] + k^S_{-\mathrm{f}}[\mathrm{W}])} \times K^{\mathrm{a}}_{\mathrm{s}} \,. \tag{8}$$

Pleasingly, by multiplying equations (7) and (8) with the aim of quantifying the overall bias towards clockwise rotation, one gets precisely the ratcheting constant K_{r} as defined in equation (5) in the main text. We stress that the above reasoning is heuristic and should not be considered as a rigorous derivation. Nevertheless, it provides an intuitive connection between the Curtin-Hammett principle and kinetic asymmetry, showing that the two conceptual frameworks are closely related.

2 Algebraic manipulation of the ratcheting constant K_{r}

To ensure that the chemomechanical cycle in Figure 2a is thermodynamically consistent, the rate constants must satisfy the constraints dictated by microscopic reversibility at equilibrium, also known as Wegscheider's conditions.[4,5] These dictate that the product of the forward rate constants along each independent cyclic pathway of reactions in the network, with neither net consumption nor net production of chemostatted species, must equal the product of the corresponding backward rate constants:

$$\frac{k^S_{+\mathrm{f}}}{k^S_{-\mathrm{f}}} \times \frac{k^R_{-\mathrm{f}}}{k^R_{+\mathrm{f}}} \times K^{\mathrm{d}}_{\mathrm{s}} \times K^{\mathrm{a}}_{\mathrm{s}} = 1 \,, \tag{9a}$$

$$\frac{k^R_{+\mathrm{h}}}{k^R_{-\mathrm{h}}} \times \frac{k^S_{-\mathrm{h}}}{k^S_{+\mathrm{h}}} \times K^{\mathrm{d}}_{\mathrm{s}} \times K^{\mathrm{a}}_{\mathrm{s}} = 1 \,. \tag{9b}$$

Furthermore, the rate constants of the anhydride formation and the hydrolysis reactions are related to the driving force:

$$\frac{[\mathrm{F}]k^{S}_{+\mathrm{f}}}{[\mathrm{W}]k^{S}_{-\mathrm{f}}} \times \frac{[\mathrm{H_2O}]k^{S}_{+\mathrm{h}}}{k^{S}_{-\mathrm{h}}} = \frac{[\mathrm{F}]k^{R}_{+\mathrm{f}}}{[\mathrm{W}]k^{R}_{-\mathrm{f}}} \times \frac{[\mathrm{H_2O}]k^{R}_{+\mathrm{h}}}{k^{R}_{-\mathrm{h}}} = e^{\Delta\mu/RT}\,. \tag{10}$$

The above equations can be used to derive equation (7) in the main text starting from equation (5) in the main text, indeed:

$$\begin{aligned}
K_\mathrm{r} =& \frac{([\mathrm{F}]k^{S}_{+\mathrm{f}} + k^{S}_{-\mathrm{h}})}{([\mathrm{F}]k^{R}_{+\mathrm{f}} + k^{R}_{-\mathrm{h}})} \times \frac{([\mathrm{W}]k^{R}_{-\mathrm{f}} + [\mathrm{H_2O}]k^{R}_{+\mathrm{h}})}{([\mathrm{W}]k^{S}_{-\mathrm{f}} + [\mathrm{H_2O}]k^{S}_{+\mathrm{h}})} \times K^{\mathrm{d}}_{\mathrm{s}} \times K^{\mathrm{a}}_{\mathrm{s}} \\
=& \frac{k^{S}_{+\mathrm{f}}}{k^{S}_{-\mathrm{f}}} \times \frac{k^{R}_{-\mathrm{f}}}{k^{R}_{+\mathrm{f}}} \times K^{\mathrm{d}}_{\mathrm{s}} \times K^{\mathrm{a}}_{\mathrm{s}} \times \frac{(1 + \frac{k^{S}_{-\mathrm{h}}}{[\mathrm{F}]k^{S}_{+\mathrm{f}}})}{(1 + \frac{k^{R}_{-\mathrm{h}}}{[\mathrm{F}]k^{R}_{+\mathrm{f}}})} \times \frac{(1 + \frac{[\mathrm{H_2O}]k^{R}_{+\mathrm{h}}}{[\mathrm{W}]k^{R}_{-\mathrm{f}}})}{(1 + \frac{[\mathrm{H_2O}]k^{S}_{+\mathrm{h}}}{[\mathrm{W}]k^{S}_{-\mathrm{f}}})} \\
=& \frac{(1 + \frac{k^{S}_{-\mathrm{h}}}{[\mathrm{F}]k^{S}_{+\mathrm{f}}})}{(1 + \frac{k^{R}_{-\mathrm{h}}}{[\mathrm{F}]k^{R}_{+\mathrm{f}}})} \times \frac{(1 + \frac{k^{R}_{-\mathrm{h}}}{[\mathrm{F}]k^{R}_{+\mathrm{f}}} e^{\Delta\mu/RT})}{(1 + \frac{k^{S}_{-\mathrm{h}}}{[\mathrm{F}]k^{S}_{+\mathrm{f}}} e^{\Delta\mu/RT})}
\end{aligned} \tag{11}$$

To derive equation (6) in the main text, we first carry out all the products in equation (11)

$$K_\mathrm{r} = \frac{1 + \frac{k^{S}_{-\mathrm{h}}}{[\mathrm{F}]k^{S}_{+\mathrm{f}}} + \frac{k^{R}_{-\mathrm{h}}}{[\mathrm{F}]k^{R}_{+\mathrm{f}}} \times e^{\Delta\mu/RT} + \frac{k^{S}_{-\mathrm{h}}k^{R}_{-\mathrm{h}}}{[\mathrm{F}]^2 k^{S}_{+\mathrm{f}}k^{R}_{+\mathrm{f}}} \times e^{\Delta\mu/RT}}{1 + \frac{k^{S}_{-\mathrm{h}}}{[\mathrm{F}]k^{S}_{+\mathrm{f}}} \times e^{\Delta\mu/RT} + \frac{k^{R}_{-\mathrm{h}}}{[\mathrm{F}]k^{R}_{+\mathrm{f}}} + \frac{k^{S}_{-\mathrm{h}}k^{R}_{-\mathrm{h}}}{[\mathrm{F}]^2 k^{S}_{+\mathrm{f}}k^{R}_{+\mathrm{f}}} \times e^{\Delta\mu/RT}}\,, \tag{12}$$

and then we multiply both the numerator and the denominator of equation (12) by $[\mathrm{F}]k^{S}_{+\mathrm{f}}/k^{S}_{-\mathrm{h}}$ to get

$$K_\mathrm{r} = \frac{\frac{[\mathrm{F}]k^{S}_{+\mathrm{f}}}{k^{S}_{-\mathrm{h}}} + 1 + \frac{k^{R}_{-\mathrm{h}}k^{S}_{+\mathrm{f}}}{k^{R}_{+\mathrm{f}}k^{S}_{-\mathrm{h}}} \times e^{\Delta\mu/RT} + \frac{k^{R}_{-\mathrm{h}}}{[\mathrm{F}]k^{R}_{+\mathrm{f}}} \times e^{\Delta\mu/RT}}{\frac{[\mathrm{F}]k^{S}_{+\mathrm{f}}}{k^{S}_{-\mathrm{h}}} + e^{\Delta\mu/RT} + \frac{k^{R}_{-\mathrm{h}}k^{S}_{+\mathrm{f}}}{k^{R}_{+\mathrm{f}}k^{S}_{-\mathrm{h}}} + \frac{k^{R}_{-\mathrm{h}}}{[\mathrm{F}]k^{R}_{+\mathrm{f}}} \times e^{\Delta\mu/RT}}\,. \tag{13}$$

Finally, by using equation (9b) to identify the Curtin-Hammett asymmetry factor as

$$\frac{k^{R}_{-\mathrm{h}}k^{S}_{+\mathrm{f}}}{k^{R}_{+\mathrm{f}}k^{S}_{-\mathrm{h}}} = \frac{k^{S}_{+\mathrm{f}}k^{R}_{+\mathrm{h}}}{k^{R}_{+\mathrm{f}}k^{S}_{+\mathrm{h}}} \times K^{\mathrm{d}}_{\mathrm{s}} \times K^{\mathrm{a}}_{\mathrm{s}} = F_{\text{C-H}}\,, \tag{14}$$

we obtain equation (6) in the main text. By computing the difference between the numerator and the denominator of equation (6) in the main text (equation (13)), we get

$$(F_{\text{C-H}} - 1) \times \left(e^{\Delta\mu/RT} - 1\right) \tag{15}$$

proving that, provided that the driving force is not null ($\Delta\mu > 0$), the cycling direction of the motor is controlled by the Curtin-Hammett asymmetry factor $F_{\text{C-H}}$. Note that equation (6) in the main text and $F_{\text{C-H}}$ are equivalent to equation (6) and the quantity expressed by "q" in a preceding work by Astumian,[6] respectively. However, in that article, connection of the "q" quantity to the Curtin-Hammett principle and the magnitude of power strokes was not recognized.

Equation (13) is derived without any assumption on the value of $\Delta\mu$, meaning that it is valid for any regime of operation (i.e., it gives the ratio between the average number of forward to backward cycles at steady states for any value of $\Delta\mu$). Provided that the motor's design is not changed and $F_{\text{C-H}}$ stays larger than one, one can appreciate from equation (15) that K_{r} is going to be larger than one if $\Delta\mu > 0$ (as discussed in the main text), equal to one if $\Delta\mu = 0$ (equilibrium), and smaller than one if $\Delta\mu < 0$. The latter regime is not explicitly discussed in the text, and would correspond to the motor cycling backward by using urea as a fuel. However, such a backward cycling would involve steps with very small rate constants such as those for backward anhydride formation and hydrolysis. As a consequence, the performance of the motor in the $\Delta\mu < 0$ scenario would be terrible in terms of cycling rate, and we did not discuss it in the main text. From equation (13), one can also appreciate that if $\Delta\mu > 0$ and $F_{\text{C-H}} < 1$ the motor will run backward but fuel will still be consumed according to the free energy gradient $\Delta\mu$. Such a behaviour is typical of the so-called weak coupled systems, i.e. systems where the direction of motion is decoupled from the direction of the fuel-to-waste reaction. Instead, in the so-called strongly coupled systems, not only the direction of motion can be inverted by changing the sign of $\Delta\mu$, but

running backward always implies fuel-production even if $\Delta\mu > 0$ in the forward direction.[7]

3 K_r for kinesin

To ensure that the chemomecanical cycle in Figure 5b is thermodynamically consistent, the rate constants must satisfy the following constraint dictated by microscopic reversibility at equilibrium:

$$\frac{k^\mathrm{F}_{-\mathrm{D}}}{k^\mathrm{F}_{+\mathrm{D}}} \times \frac{k^\mathrm{B}_{+\mathrm{h}}}{k^\mathrm{B}_{-\mathrm{h}}} \times \frac{k^\mathrm{F}_{+\mathrm{T}}}{k^\mathrm{F}_{-\mathrm{T}}} \times \frac{k^\mathrm{B}_{-\mathrm{T}}}{k^\mathrm{B}_{+\mathrm{T}}} \times \frac{k^\mathrm{F}_{-\mathrm{h}}}{k^\mathrm{F}_{+\mathrm{h}}} \times \frac{k^\mathrm{B}_{+\mathrm{D}}}{k^\mathrm{B}_{-\mathrm{D}}} \times K_\mathrm{s}^2 = 1 \tag{16}$$

Furthermore, the rate constants of the chemical processes fulfilling kinesin-catalyzed ATP-hydrolysis are related to the driving force by the following relation:

$$\frac{k^\mathrm{F}_{-\mathrm{D}}}{[\mathrm{ADP}]k^\mathrm{F}_{+\mathrm{D}}} \times \frac{[\mathrm{H_2O}]k^\mathrm{B}_{+\mathrm{h}}}{[\mathrm{P_i}]k^\mathrm{B}_{-\mathrm{h}}} \times \frac{[\mathrm{ATP}]k^\mathrm{F}_{+\mathrm{T}}}{k^\mathrm{F}_{-\mathrm{T}}} \times \frac{k^\mathrm{B}_{-\mathrm{D}}}{[\mathrm{ADP}]k^\mathrm{B}_{+\mathrm{D}}} \times \frac{[\mathrm{H_2O}]k^\mathrm{F}_{+\mathrm{h}}}{[\mathrm{P_i}]k^\mathrm{F}_{-\mathrm{h}}} \times \frac{[\mathrm{ATP}]k^\mathrm{B}_{+\mathrm{T}}}{k^\mathrm{B}_{-\mathrm{T}}} = e^{2\Delta\mu_\mathrm{ATP}/RT}, \tag{17}$$

where we introduced $\Delta\mu_\mathrm{ATP} = \mu_\mathrm{ATP} + \mu_\mathrm{H_2O} - \mu_\mathrm{ADP} - \mu_\mathrm{P_i}$. To evaluate the kinetic asymmetry of the chemomecanical cycle in Figure 5b at the steady state, namely, the ratio between the overall cycling frequencies in the forward and backward directions, we compute the kinesin's ratcheting constant $K_\mathrm{r}^{(\mathrm{kin})}$ analogously to what we have done in the main text for the autonomous single bond rotary motor fueled by carbodiimide hydration. By considering the 4 possible forward cycles and their reversals, and by simplifying the common factor involving the concentrations of kinesin's species, we obtain

$$K_\mathrm{r}^{(\mathrm{kin})} = K_\mathrm{s}^2 \times \frac{k^\mathrm{F}_{-\mathrm{D}}\tilde{k}^\mathrm{B}_{+\mathrm{h}}\tilde{k}^\mathrm{F}_{+\mathrm{T}}k^\mathrm{F}_{-\mathrm{D}}\tilde{k}^\mathrm{B}_{+\mathrm{h}}\tilde{k}^\mathrm{F}_{+\mathrm{T}} + k^\mathrm{F}_{-\mathrm{D}}\tilde{k}^\mathrm{B}_{+\mathrm{h}}\tilde{k}^\mathrm{F}_{+\mathrm{T}}k^\mathrm{B}_{-\mathrm{T}}\tilde{k}^\mathrm{F}_{-\mathrm{h}}\tilde{k}^\mathrm{B}_{+\mathrm{D}} + k^\mathrm{B}_{-\mathrm{T}}\tilde{k}^\mathrm{F}_{-\mathrm{h}}\tilde{k}^\mathrm{B}_{+\mathrm{D}}k^\mathrm{F}_{-\mathrm{D}}\tilde{k}^\mathrm{B}_{+\mathrm{h}}\tilde{k}^\mathrm{F}_{+\mathrm{T}} + k^\mathrm{B}_{-\mathrm{T}}\tilde{k}^\mathrm{F}_{-\mathrm{h}}\tilde{k}^\mathrm{B}_{+\mathrm{D}}k^\mathrm{B}_{-\mathrm{T}}\tilde{k}^\mathrm{F}_{-\mathrm{h}}\tilde{k}^\mathrm{B}_{+\mathrm{D}}}{\tilde{k}^\mathrm{F}_{+\mathrm{D}}\tilde{k}^\mathrm{B}_{-\mathrm{h}}k^\mathrm{F}_{-\mathrm{T}}\tilde{k}^\mathrm{F}_{+\mathrm{D}}\tilde{k}^\mathrm{B}_{-\mathrm{h}}k^\mathrm{F}_{-\mathrm{T}} + \tilde{k}^\mathrm{F}_{+\mathrm{D}}\tilde{k}^\mathrm{B}_{-\mathrm{h}}k^\mathrm{F}_{-\mathrm{T}}\tilde{k}^\mathrm{B}_{+\mathrm{T}}\tilde{k}^\mathrm{F}_{+\mathrm{h}}k^\mathrm{B}_{-\mathrm{D}} + \tilde{k}^\mathrm{B}_{+\mathrm{T}}\tilde{k}^\mathrm{F}_{+\mathrm{h}}k^\mathrm{B}_{-\mathrm{D}}\tilde{k}^\mathrm{F}_{+\mathrm{D}}\tilde{k}^\mathrm{B}_{-\mathrm{h}}k^\mathrm{F}_{-\mathrm{T}} + \tilde{k}^\mathrm{B}_{+\mathrm{T}}\tilde{k}^\mathrm{F}_{+\mathrm{h}}k^\mathrm{B}_{-\mathrm{D}}\tilde{k}^\mathrm{B}_{+\mathrm{T}}\tilde{k}^\mathrm{F}_{+\mathrm{h}}k^\mathrm{B}_{-\mathrm{D}}}, \tag{18}$$

where, for compactness of notation, we introduced pseudo-first order rate constants incorporating the concentration of the chemostatted species: $\tilde{k}^\mathrm{F/B}_{+\mathrm{T}} = [\mathrm{ATP}]k^\mathrm{F/B}_{+\mathrm{T}}$, $\tilde{k}^\mathrm{F/B}_{+\mathrm{h}} = [\mathrm{H_2O}]k^\mathrm{F/B}_{+\mathrm{h}}$,

$\tilde{k}_{-\mathrm{h}}^{\mathrm{F/B}} = [\mathrm{P_i}]k_{-\mathrm{h}}^{\mathrm{F/B}}$, and $\tilde{k}_{+\mathrm{D}}^{\mathrm{F/B}} = [\mathrm{ADP}]k_{+\mathrm{D}}^{\mathrm{F/B}}$. By exploiting equation (16), the ratcheting constant for the kinesin's chemomechanical cycle in equation (18) can be rewritten in a form analogous to the one in equation (7) in the main text:

$$K_{\mathrm{r}}^{(\mathrm{kin})} = \frac{\left(1 + \frac{k_{-\mathrm{T}}^{\mathrm{B}}[\mathrm{P_i}]k_{-\mathrm{h}}^{\mathrm{F}}[\mathrm{ADP}]k_{+\mathrm{D}}^{\mathrm{B}}}{[\mathrm{ATP}]k_{+\mathrm{T}}^{\mathrm{F}}[\mathrm{H_2O}]k_{+\mathrm{h}}^{\mathrm{B}}k_{-\mathrm{D}}^{\mathrm{F}}}\right)\left(1 + \frac{[\mathrm{ATP}]k_{+\mathrm{T}}^{\mathrm{F}}[\mathrm{H_2O}]k_{+\mathrm{h}}^{\mathrm{B}}k_{-\mathrm{D}}^{\mathrm{F}}}{k_{-\mathrm{T}}^{\mathrm{B}}[\mathrm{P_i}]k_{-\mathrm{h}}^{\mathrm{F}}[\mathrm{ADP}]k_{+\mathrm{D}}^{\mathrm{B}}}\right)}{\left(1 + \frac{[\mathrm{ATP}]k_{+\mathrm{T}}^{\mathrm{B}}[\mathrm{H_2O}]k_{+\mathrm{h}}^{\mathrm{F}}k_{-\mathrm{D}}^{\mathrm{B}}}{k_{-\mathrm{T}}^{\mathrm{F}}[\mathrm{P_i}]k_{-\mathrm{h}}^{\mathrm{B}}[\mathrm{ADP}]k_{+\mathrm{D}}^{\mathrm{F}}}\right)\left(1 + \frac{k_{-\mathrm{T}}^{\mathrm{F}}[\mathrm{P_i}]k_{-\mathrm{h}}^{\mathrm{B}}[\mathrm{ADP}]k_{+\mathrm{D}}^{\mathrm{F}}}{[\mathrm{ATP}]k_{+\mathrm{T}}^{\mathrm{B}}[\mathrm{H_2O}]k_{+\mathrm{h}}^{\mathrm{F}}k_{-\mathrm{D}}^{\mathrm{B}}}\right)}, \tag{19}$$

which only contains ratios of chemical rate constants, thus highlighting the necessity of a certain degree of chemical gating at the level of either the forward or backward chemical reactions for the kinesin to walk directionally.[6] However, by using equation (17) along with some algebraic passages similar to the ones employed in Supplementary Section II to go from equation (11) to equation (6) in the main text, we recover an equivalent expression which is similar to the latter:

$$K_{\mathrm{r}}^{(\mathrm{kin})} = \frac{\gamma^{(\mathrm{kin})} + 1 + \left(\frac{k_{+\mathrm{T}}^{\mathrm{F}}k_{+\mathrm{h}}^{\mathrm{B}}k_{-\mathrm{D}}^{\mathrm{F}}}{k_{+\mathrm{T}}^{\mathrm{B}}k_{+\mathrm{h}}^{\mathrm{F}}k_{-\mathrm{D}}^{\mathrm{B}}}K_{\mathrm{s}}\right)^2 e^{2\Delta\mu_{\mathrm{ATP}}/RT}}{\gamma^{(\mathrm{kin})} + e^{2\Delta\mu_{\mathrm{ATP}}/RT} + \left(\frac{k_{+\mathrm{T}}^{\mathrm{F}}k_{+\mathrm{h}}^{\mathrm{B}}k_{-\mathrm{D}}^{\mathrm{F}}}{k_{+\mathrm{T}}^{\mathrm{B}}k_{+\mathrm{h}}^{\mathrm{F}}k_{-\mathrm{D}}^{\mathrm{B}}}K_{\mathrm{s}}\right)^2},$$
$$\text{with} \quad \gamma^{(\mathrm{kin})} = \frac{[\mathrm{ATP}]k_{+\mathrm{T}}^{\mathrm{F}}[\mathrm{H_2O}]k_{+\mathrm{h}}^{\mathrm{B}}k_{-\mathrm{D}}^{\mathrm{F}}}{k_{-\mathrm{T}}^{\mathrm{B}}[\mathrm{P_i}]k_{-\mathrm{h}}^{\mathrm{F}}[\mathrm{ADP}]k_{+\mathrm{D}}^{\mathrm{B}}} + \frac{k_{-\mathrm{T}}^{\mathrm{F}}[\mathrm{P_i}]k_{-\mathrm{h}}^{\mathrm{B}}[\mathrm{ADP}]k_{+\mathrm{D}}^{\mathrm{F}}}{[\mathrm{ATP}]k_{+\mathrm{T}}^{\mathrm{B}}[\mathrm{H_2O}]k_{+\mathrm{h}}^{\mathrm{F}}k_{-\mathrm{D}}^{\mathrm{B}}}e^{2\Delta\mu_{\mathrm{ATP}}/RT}. \tag{20}$$

The above equation shows that, in the absence of a power source (i.e., $\Delta\mu_{\mathrm{ATP}} = 0$), the value of $K_{\mathrm{r}}^{(\mathrm{kin})}$ is unavoidably one, preventing the motor from cycling directionally at equilibrium. Provided that $\Delta\mu_{\mathrm{ATP}} \neq 0$, the factor

$$\frac{k_{+\mathrm{T}}^{\mathrm{F}}k_{+\mathrm{h}}^{\mathrm{B}}k_{-\mathrm{D}}^{\mathrm{F}}}{k_{+\mathrm{T}}^{\mathrm{B}}k_{+\mathrm{h}}^{\mathrm{F}}k_{-\mathrm{D}}^{\mathrm{B}}}K_{\mathrm{s}} \tag{21}$$

dictates the directionality as discussed in the main text.

References

(1) Seeman, J. I. Effect of conformational change on reactivity in organic chemistry. Evaluations, applications, and extensions of Curtin-Hammett Winstein-Holness kinetics. *Chem. Rev.* **1983**, *83*, 83–134.

(2) Seeman, J. I. The Curtin-Hammett principle and the Winstein-Holness equation: new definition and recent extensions to classical concepts. *J. Chem. Educ.* **1986**, *63*, 42.

(3) Andraos, J. Quantification and Optimization of Dynamic Kinetic Resolution. *J. Phys. Chem. A* **2003**, *107*, 2374–2387.

(4) Rao, R.; Esposito, M. Nonequilibrium Thermodynamics of Chemical Reaction Networks: Wisdom from Stochastic Thermodynamics. *Phys. Rev. X* **2016**, *6*, 041064.

(5) Astumian, R. D. Kinetic asymmetry allows macromolecular catalysts to drive an information ratchet. *Nat. Commun.* **2019**, *10*, 3837.

(6) Astumian, R. D. Irrelevance of the Power Stroke for the Directionality, Stopping Force, and Optimal Efficiency of Chemically Driven Molecular Machines. *Biophys. J.* **2015**, *108*, 291–303.

(7) Borsley, S.; Leigh, D. A.; Roberts, B. M. W. Chemical fuels for molecular machinery. *Nat. Chem.* **2022**, *14*, 728–738.
